# Long-Term Complete Response and Survival in Metastatic Extramammary Paget’s Disease Treated With Trastuzumab Plus Paclitaxel: A Case Report

**DOI:** 10.7759/cureus.58924

**Published:** 2024-04-24

**Authors:** Mario Giovanni Chilelli, Carlo Signorelli, Julio Rodrigo Giron Berrios, Armando Raso, Fabrizio Nelli, Enzo Maria Ruggeri

**Affiliations:** 1 Department of Oncology and Hematology, Medical Oncology Unit, Belcolle Hospital, ASL Viterbo, Viterbo, ITA; 2 Department of Radiology, Radiology Unit, Belcolle Hospital, ASL Viterbo, Viterbo, ITA

**Keywords:** target therapy, chemotherapy, scrotum, extramammary paget’s disease, case report

## Abstract

Extramammary Paget’s disease is a rare skin cancer that usually arises from the secretory cells of the apocrine glands. In most cases, an extramammary Paget’s tumor occurs as a single intraepithelial form not associated with another cancer, although rarely, it may be associated with other loco-regional or distant cancer. It is generally slow-growing and diagnosed in situ. Most often, surgical excision with wide margins is curative, with the local recurrence rate being lower after the Mohs micrographic surgery technique. Nonetheless, relapses are frequent. In the metastatic setting, there are no treatment guidelines or standard therapies; additionally, the experience is limited to a few individual cases, and the efficacy of conventional chemotherapies is not well-defined. Moreover, chemotherapy can also have serious side effects; therefore, there is a need to identify more effective and less toxic therapies. In this case report, we have observed a long-lasting complete response with anti-HER2 plus paclitaxel.

## Introduction

Extramammary Paget’s disease (EMPD) is a rare skin cancer that usually arises from the secretory cell of the apocrine glands; the most frequent location is the vulva, perianal region, perineal, scrotum, and penis [1], but occurrence in areas without apocrine glands has been described [2]. In 2012, a crude incidence rate and an age-standardized incidence rate of 0.7 and 0.6 per 1,000,000 person-years, respectively, were described in Europe [3]; however, the true incidence of the disease is still unknown. Additionally, in the Caucasian population, it is more common in older females, and the peak is between 60 to 70 years of age [4]; however, in the Asian population, a male predominance was reported [5]. It usually presents as an erythematous or eczematous plaque with well-defined borders and slow growth [6]. Patients often complain of itching and pain [4]. Histologically, it is characterized by the presence of Paget’s cells, which are large cells with prominent nucleoli, abundant eosinophilic cytoplasm, and occasionally cytoplasmic clearing. In most cases, extramammary Paget’s tumor occurs as a single intraepithelial form (primary EMPD) but may be associated in over 30% of patients with an underlying locoregional or distant cancer (secondary EMPD) [7]. It is generally slow-growing and diagnosed in situ. Most often, surgical excision with wide margins is curative, with the local recurrence rate being lower after the Mohs micrographic surgery technique. Nonetheless, relapses are frequent [8]. On the contrary, when the tumor invades the dermis, a condition that can occur even after years to the principal diagnosis, the prognosis becomes poor, as it frequently metastasizes to lymph nodes, with over one-third of these patients developing distant metastasis [9]. In the metastatic setting, there are no treatment guidelines or standard therapies; additionally, the experience is limited to a few individual cases, and the efficacy of conventional chemotherapies is not well-defined.

## Case presentation

The purpose of this case report derives from the lack of guidelines and the absence of standard therapies for metastatic disease, as well as the long-lasting complete response obtained with targeted therapy in this particular case.

**Figure 1:**
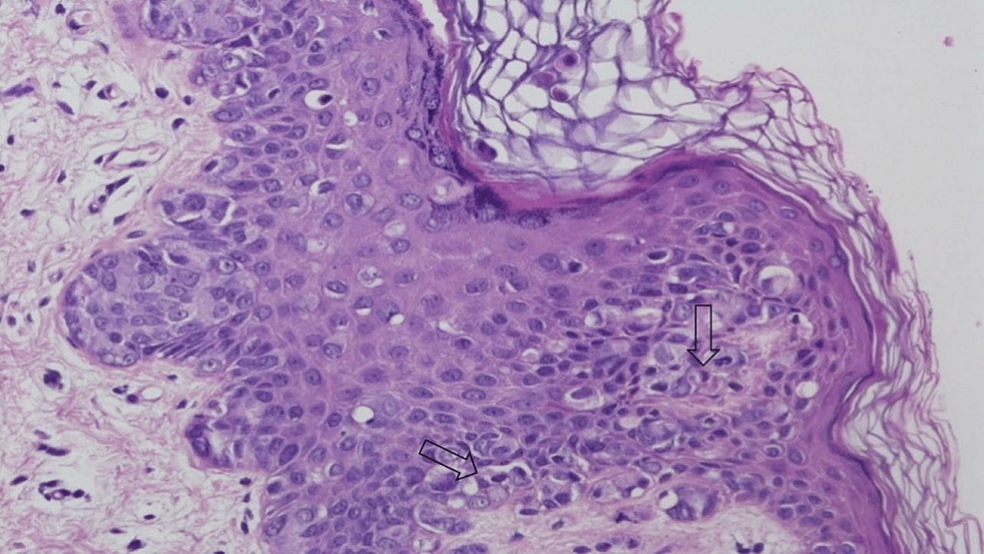
Invasive EMPD Intraepidermal proliferation of predominantly single cells with admixed nests throughout the epidermis; Paget cells are large, with ample amphophilic cytoplasm, a large nucleus, and prominent nucleoli. EMPD: extramammary Paget’s disease

**Figure 2:**
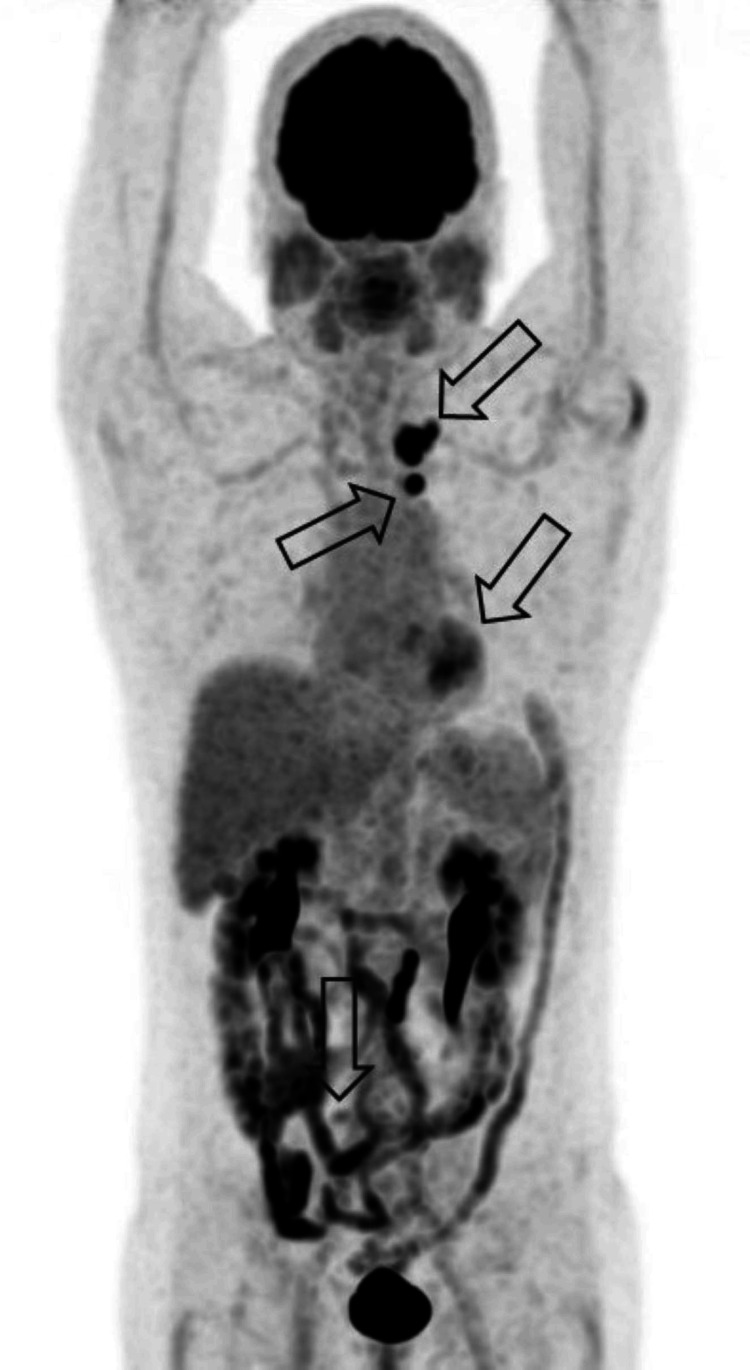
18-FDG PET scan performed before the treatment showed recurrence in the left retroclavicular lymph, left upper mediastinum, para-aortic, and lumbo-aortic nodes 18-FDG: fludeoxyglucose F18; PET: positron emission tomography

**Figure 3:**
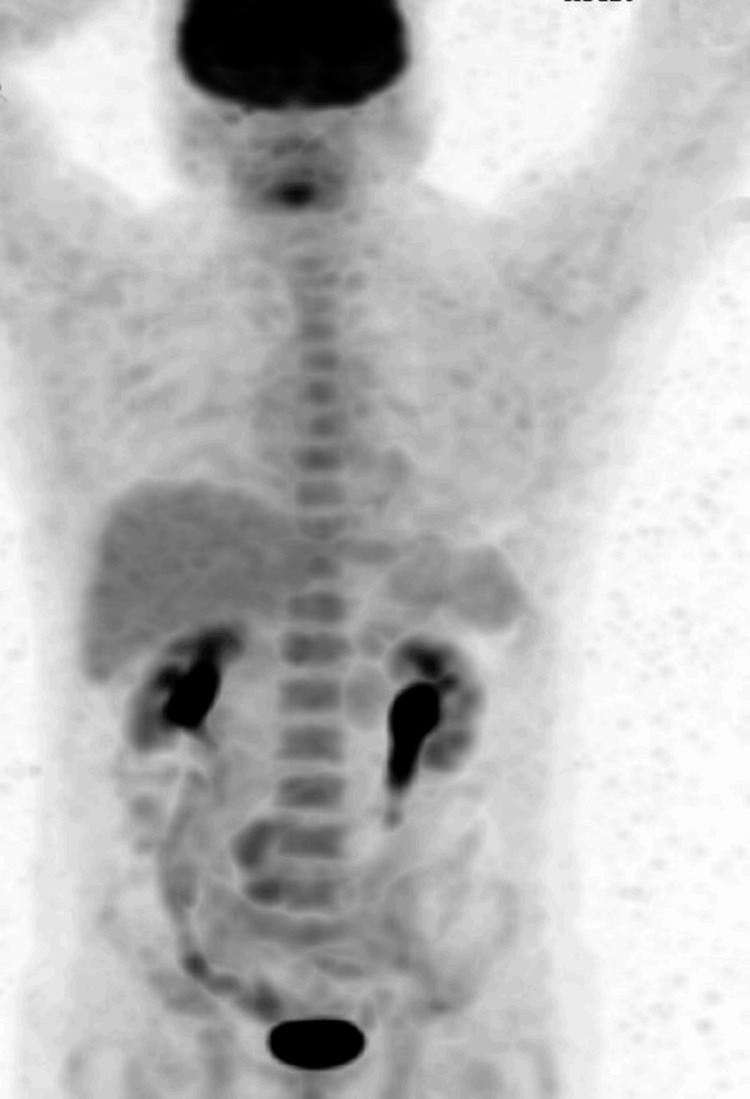
18-FDG PET scan after 11 weeks of therapy was negative for oncological disease (complete response) 18-FDG: fludeoxyglucose F18; PET: positron emission tomography

A 57-year-old white man in good general condition came to our observation after surgical removal of a small erythematous plaque from the scrotum in October 2018. The anamnesis showed hypertension, transient cerebral ischemia, epilepsy, and surgery to close the Botallo duct. The histological report of the scrotum revealed carcinoma of the apocrine adnexal glands, ulcerated, intraepidermal, and infiltrating the papillary dermis, with a maximum thickness of 7 mm (extramammary Paget’s disease). Angioinvasion is reported. The lesion extends to the longitudinal excision margin (Figure 1). The wide local excision surgery performed two months later revealed a residual disease of 6 mm. The patient’s physical examination was negative, so the patient began follow-up. In March 2019, the CT scan showed recurrence in the right iliac obturator and inguinal lymph nodes. The positron emission tomography (PET)/computed tomography (CT) scan was positive in the same sites. Subsequently, metastasis from EMPD was confirmed by fine-needle aspiration and the patient underwent iliac-obturator lymphadenectomy. Metastases from EMPD without extracapsular spread were found in 6 out of 13 lymph nodes removed, 1 of which showed massive metastasis. Afterward, follow-up was negative until August 2020, when a PET/CT scan showed recurrence in left retroclavicular lymph, left upper mediastinum, para-aortic, and left lumbo-aortic nodes (Figure 2). Fine-needle aspiration of the left supraclavicular lymph node confirmed metastases from EMPD. HER-2 expression was positive (HER2 + 3 DAKO score) while mismatch repair was proficient (pMMR). So it was decided to submit the patient to chemotherapy and anti-HER2 treatment. In November 2020, the patient started treatment with weekly paclitaxel (80 mg/m²) plus trastuzumab (4 mg/kg loading dose and 2 mg/kg for subsequent administrations). After 11 weeks of treatment, a PET/CT showed a clinical complete response (RC) (Figure 3). The PET/CT showed a slight gastric uptake due to gastritis, as evidenced by gastroscopy performed a few days later. 

**Figure 4:**
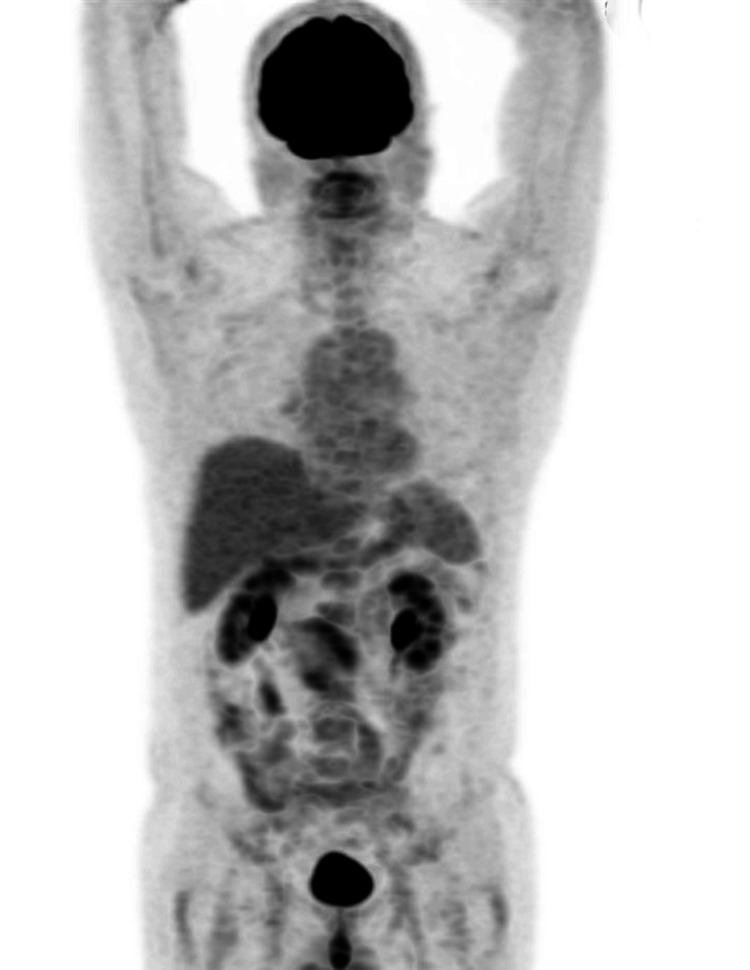
18-FDG PET scan performed in January 2024 was negative for oncological disease (complete response) 18-FDG: fludeoxyglucose F18; PET: positron emission tomography

Treatment was continued with the same schedule for six more weeks and then with only trastuzumab 6 mg/kg given every three weeks. Complete clinical response was maintained at the latest PET/CT scan performed on January 29, 2024 (Figure 4). Three-weekly trastuzumab treatment is still ongoing.

## Discussion

In metastatic EMPD, therapeutic experience is limited and there are no standard therapies. Overall survival (OS), moreover, is only 16% at five years [10]. The efficacy of conventional chemotherapies is limited. The various regimens of chemotherapy used, including PET (cisplatin-epirubicin-paclitaxel), FECOM (carboplatin, epirubicin, vincristine, mitomycin C), low-dose FP (low-dose 5-fluorouracil/cisplatin), and docetaxel monotherapy, have produced, in small case series, a partial remission of the disease in 50-80% of cases and rarely complete response. However, the duration of response with these drug regimens is short, with a median progression-free survival (PFS) ranging from 5.2 to 8 months, and a median OS ranging from 9.4 to 20.1 months [11-14]. Additionally, chemotherapy can also have serious side effects. For example, Hirai et al. reported grade 3-4 toxicities in four out of five patients with the modified PET regimen [11].

PD-L1/L2 expression and the presence of MSI-H (microsatellite instability-high) status, predictors of tumor response to immune checkpoint inhibitors, are generally absent in extramammary Paget’s disease and the role of immunotherapy is yet to be defined. However, a case with a durable response was recently reported with the combination of ipilimumab and nivolumab in a patient with an absence of expression of PD-L1 and PD-L2 by tumor cells and minimal PD-L1 expression in the tumor microenvironment [15].

It has also been hypothesized that androgen blockade therapy would appear to have therapeutic potential for EMPD with AR expression [16]. In a case report of the vulva, trastuzumab used as monotherapy in HER-2 overexpressing tumors has shown some activity with PFS of about 12 months [17], and a complete remission in an extramammary Paget’s disease of the scrotum has been reported [18]. Published case reports of patients treated with a combination of trastuzumab plus paclitaxel have already shown efficacy, with a PFS ranging from 13 to 30 months [19,20] and an OS of 25-30 months [21,22]. This data suggests that HER-2 overexpression has a role in the pathogenesis and progression of a subset of metastatic EMPD, and at the same time, suggests the possibility that HER-2 blockade could be an effective therapy.

Therefore, based on this information, we submit the patient to weekly trastuzumab plus paclitaxel, a known effective regimen in HER-2 positive breast cancer, assuming that the “molecularly targeted” therapy could also be effective in our case. Indeed, the response to treatment was optimal with a complete response after 11 weeks of therapy, still maintained after 38 months. Treatment toxicity resulted in G3 neutropenia at week 3 with omission of paclitaxel at week 3 and a 20% dose reduction of paclitaxel for subsequent administrations.

## Conclusions

The absence of guidelines and standard therapies in metastatic EMPD should encourage participation in clinical trials. In HER-2 overexpressing disease (HER-2 3+ or HER-2 2+ FISH positive), targeted therapy with trastuzumab plus paclitaxel appears to be effective and could be the therapy of choice in this setting. The experience gained in the treatment of HER-2-positive breast cancer suggests that the toxicity of this treatment is well manageable.

Gene sequencing with next-generation sequencing (NGS) is recommended to identify additional molecular targets and expand the limited therapeutic availability in this malignancy.
